# Regulation of CCR4-NOT complex deadenylase activity and cellular responses by MK2-dependent phosphorylation of CNOT2

**DOI:** 10.1080/15476286.2021.2021676

**Published:** 2022-02-06

**Authors:** Toru Suzuki, Miyuki Hoshina, Saori Nishijima, Naosuke Hoshina, Chisato Kikuguchi, Takumi Tomohiro, Akira Fukao, Toshinobu Fujiwara, Tadashi Yamamoto

**Affiliations:** a Laboratory for Immunogenetics, Center for Integrative Medical Sciences, Riken, Yokohama, Japan; b Cell Signal Unit, Okinawa Institute of Science and Technology Graduate University, Onna, Japan; c Laboratory of Biochemistry, Kindai University, Higashi-Osaka, Japan

**Keywords:** CCR4-NOT complex, MK2, mRNA decay, phosphorylation, stress response

## Abstract

CCR4-NOT complex-mediated mRNA deadenylation serves critical functions in multiple biological processes, yet how this activity is regulated is not fully understood. Here, we show that osmotic stress induces MAPKAPK-2 (MK2)-mediated phosphorylation of CNOT2. Programmed cell death is greatly enhanced by osmotic stress in CNOT2-depleted cells, indicating that CNOT2 is responsible for stress resistance of cells. Although wild-type (WT) and non-phosphorylatable CNOT2 mutants reverse this sensitivity, a phosphomimetic form of CNOT2, in which serine at the phosphorylation site is replaced with glutamate, does not have this function. We also show that mRNAs have elongated poly(A) tails in CNOT2-depleted cells and that introduction of CNOT2 WT or a non-phosphorylatable mutant, but not phosphomimetic CNOT2, renders their poly(A) tail lengths comparable to those in control HeLa cells. Consistent with this, the CCR4-NOT complex containing phosphomimetic CNOT2 exhibits less deadenylase activity than that containing CNOT2 WT. These data suggest that CCR4-NOT complex deadenylase activity is regulated by post-translational modification, yielding dynamic control of mRNA deadenylation.

## Introduction

Living organisms are repeatedly exposed to a wide variety of extrinsic and intrinsic signals, including those initiated by humoral factors, pathogens, physical damage, and somatic mutations. Rapid and appropriate cellular responses to those stimuli are crucial for cells and organisms to adapt to environmental changes. Regulation of gene expression is a central mechanism in various biological activities, including stress responses.

Gene expression is regulated both during and after transcription. One important post-transcriptional mechanism is mRNA decay, which maintains proper mRNA levels to ensure cellular and tissue homoeostasis. The usual mRNA decay pathway is initiated by shortening of poly(A) tails, called deadenylation [1]. The CCR4-NOT complex, which is conserved from yeast to humans, is central to mRNA deadenylation.

CCR4-NOT deadenylase is a multi-protein complex, consisting of at least eight subunits (Ccr4, Caf1, Caf40, Caf130, Not1, Not2, Not3, Not4) in yeast. In *S. cerevisiae* species of yeast, the complex contains two homologous molecules, Not5 and Not3, that likely originate from a gene duplication event [2]. On the other hand, the mammalian CCR4-NOT complex contains CNOT1, CNOT2, CNOT3, either CNOT7 or CNOT8, either CNOT6 or CNOT6L, CNOT9, CNOT10 and CNOT11 [2]. The deadenylases CNOT7/8 and CNOT6/6 L are orthologs of yeast Caf1 and Ccr4, respectively. Among the non-deadenylase subunits, CNOT1 functions as the scaffold of the complex. The CNOT10-CNOT11 module, the deadenylases, CNOT9, and the CNOT2-CNOT3 heterodimer bind to different domains of CNOT1 from the N-terminal to the C-terminal region [3–8]. Consistent with this function, *Cnot1*-deficiency largely prevents complex formation and deadenylase activity, impairing cell viability and tissue function [9–14]. Suppression of CNOT2 or CNOT3 using small interfering RNA (siRNA)-mediated knockdown or knockout mouse strains, also causes a decrease of deadenylase activity in various organisms [11,15–24]. Importantly, *S. cerevisiae* Not5 subunit, the ortholog of the mammalian CNOT3, is responsible for codon optimality-dependent mRNA decay by binding to the ribosomal E-site [25]. CNOT9 is involved in microRNA (miRNA)-mediated gene silencing via interactions with GW182 proteins [4]. Moreover, CNOT9/Caf40 mediates an interaction of the CCR4-NOT complex with nucleic acids and several interactors [26–29]. CNOT10 and CNOT11 are required for full deadenylase activity with potential RNA binding ability [5,30]. While various RNA-binding proteins contribute to target recognition of the CCR4-NOT complex [15,31–33], the mechanisms by which non-deadenylase subunits control CCR4-NOT deadenylase activity are not fully understood.

Extracellular stimuli such as environmental stress and inflammatory cytokines activate a signalling pathway containing Jun N-terminal kinase (JNK), p38 mitogen-activated protein kinase (MAPK), and MAPK-activated protein kinase 2 (MK2). This pathway determines levels of transcripts by regulating transcription factors and RNA-binding proteins (RBPs) [34]. In other words, these signalling mechanisms contribute to precise regulation of gene expression and cellular responses to extracellular stimuli, both transcriptionally and post-transcriptionally. Recruitment of the CCR4-NOT complex to target mRNAs is one of the important steps of gene expression control. Recruitment can be mediated by RBPs, such as zinc finger protein 36 family proteins (ZFP36, ZFP36 L1 and ZFP36 L2), which are phosphorylation targets of JNK, p38MAPK, and MK2 [35–40]. However, it is not known whether changes in the extracellular environment directly impact activity of the CCR4-NOT complex.

Here, we examine whether an extracellular stimulus-mediated signalling pathway controls the function of the CCR4-NOT complex. We find that CNOT2 is phosphorylated at multiple sites in response to various stimuli, and provide evidence that this phosphorylation affects both deadenylase activity of the CCR4-NOT complex and sensitivity to extracellular stress. Therefore, we conclude that post-translational modification dynamically regulates deadenylation-dependent mRNA decay, depending on the extracellular environment.

## Results

### Phosphorylation of CNOT2 following osmotic stress

**Figure 1.**
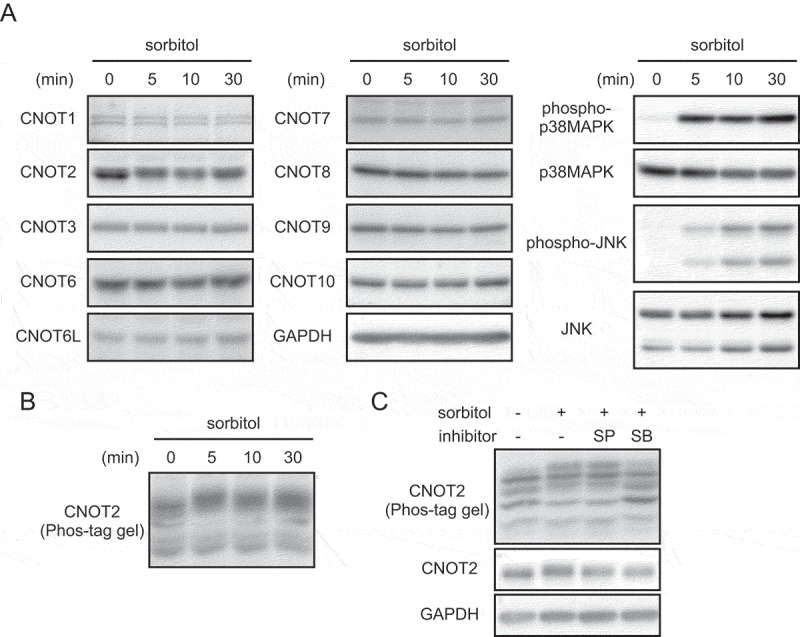
Phosphorylation of CNOT2 after osmotic stress.

To examine expression of CCR4-NOT complex subunits under extracellular stress, we treated HeLa cells with sorbitol, an osmotic stressor. We monitored activation of stress responses by detecting phosphorylation of p38MAPK and JNK (Fig. 1A). While protein levels of CCR4-NOT complex subunits hardly changed, CNOT2 protein bands migrated more slowly in an acrylamide gel after osmotic stress than before (Fig. 1A). Phos-tag SDS-polyacrylamide gel electrophoresis (PAGE) of osmotically stress-treated cell lysates followed by immunoblotting revealed that the mobility shift of CNOT2 was due to phosphorylation (Fig. 1B). When we performed immunoprecipitation using an anti-CNOT2 antibody, other CCR4-NOT complex subunits were co-immunoprecipitated in the same manner before and after osmotic stress, suggesting that osmotic stress does not alter formation of the CCR4-NOT complex (Supplementary Fig. S1).

(A) HeLa cells were treated with sorbitol. Lysates were prepared at the indicated times after treatment, and analysed by immunoblot. (B) HeLa cell lysates in (A) were separated on Phos-tag SDS-PAGE and analysed by immunoblot using anti-CNOT2 antibody. (C) HeLa cells were pretreated with inhibitors for 15 min and then treated with sorbitol for an additional 30 min. Cell lysates were separated on SDS-PAGE or Phos-tag SDS-PAGE, followed by immunoblot. SP: SP600125 (JNK inhibitor), SB: SB203580 (p38MAPK inhibitor).

### CNOT2 is phosphorylated at Ser 101 by MK2

**Figure 2.**
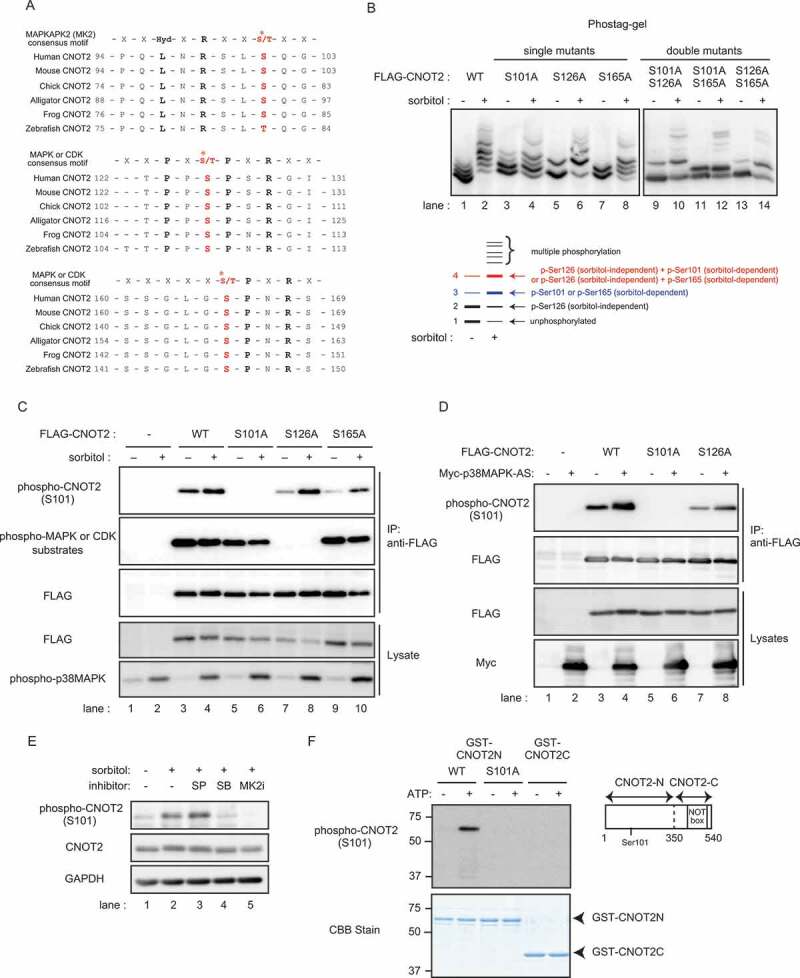
Osmotic stress-induced, MK2-dependent phosphorylation of CNOT2 at Ser101.

To identify kinases responsible for CNOT2 phosphorylation after osmotic stress, we treated HeLa cells with inhibitors of protein kinases relevant to stress responses. Immunoblot analyses showed that SB203580, an inhibitor of p38MAPK [41], but not SP600125, an inhibitor of JNKs [42], suppressed osmotic, stress-induced CNOT2 phosphorylation (Fig. 1C). Several phosphorylation sites in human CNOT2 are detected using high throughput analyses and are found in post-translational modification databases [43]. Among them, amino acid sequences surrounding Ser126 and Ser165 correspond to consensus motifs for MAPK or cyclin-dependent kinase (CDK)-dependent phosphorylation (Fig. 2A). We also found that the amino acid sequence around Ser101 corresponds to a consensus motif for MK2-dependent phosphorylation [44], though it has not been reported as a phosphorylation site (Fig. 2A). Importantly, these amino acid sequences are conserved among vertebrates (Fig. 2A). To examine whether these Ser residues in CNOT2 are phosphorylated in response to osmotic stress, we constructed expression vectors for FLAG-tagged CNOT2 WT and six mutants in which these three serine residues were replaced with alanine (single mutants S101A; S126A; S165A; double mutants S101,126A; S101,165A; S126,165A; and triple mutant S101,126,165A). We transfected those vectors into HEK293T cells, and prepared lysates before and after osmotic stress. We analysed anti-FLAG immunoprecipitates of those cell lysates using Phos-tag SDS-PAGE followed by immunoblotting. The results showed the presence of multiple bands of CNOT2 WT protein in both untreated and sorbitol-treated cells, suggesting sorbitol-independent and dependent phosphorylation. Under untreated condition, four bands were clearly visible in CNOT2 WT (Fig. 2B, lane 1; see a schematic representation of immunoblot images, labelled 1–4). Following sorbitol treatment, we observed an increased intensity of bands 3 and 4 as well as an appearance of multiple bands above band 4 (Fig. 2B, lane 2; see the schematic representation of immunoblot images). In addition, the intensity of band 1 decreased after sorbitol treatment in CNOT2 WT, while that of band 2 slightly decreased (Fig. 2B, lane 2; see the schematic representation of immunoblot images). We observed differences in the band patterns between CNOT2 WT and serine mutants described above. (1) Bands 2 and 4 disappeared in single mutant S126A before and after sorbitol treatment (Fig. 2B, lanes 5 and 6). Similar band pattens were observed in the double mutants S101,126A and S126,165A (Fig. 2B, lanes 9, 10, 13, 14), suggesting that bands 2 and 4 contains S126 phosphorylation and the phosphorylation event is independent of sorbitol treatment. (2) Four bands were present in the single mutant S101A similarly to CNOT2 WT under untreated condition (Fig. 2B, lane 3). After sorbitol treatment, the intensity of bands 3 and 4 increased, while that of band 1 decreased (Fig. 2B, lane 4). Bands 3 and 4 disappeared in the double mutant S101,165A (Fig. 2B, lanes 11 and 12), suggesting that bands 3 and 4 contain S165 phosphorylation and the phosphorylation event is augmented by sorbitol treatment. Increased intensity of band 3 in the double mutant S101,126A after sorbitol treatment further suggested that band 3 contains S165 phosphorylation (Fig. 2B, lanes 9 and 10). (3) In the single mutant S165A under untreated condition, bands 1 and 2 were detected, while bands 3 and 4 were hardly detected (Fig. 2B, lane 7). After sorbitol treatment, bands 3 and 4 became visible, while intensity of bands 1 and 2 decreased (Fig. 2B, lane 8). Bands 3 and 4 disappeared in the double mutant S101,165A (Fig. 2B, lanes 11 and 12), suggesting that bands 3 and 4 contain sorbitol-dependent S101 phosphorylation. Increase of the band 3 intensity in the double mutant S126,165A after sorbitol treatment supports the idea (Fig. 2B, lanes 13 and 14). Finally, in the triple mutant S101,126,165A, we detected the fastest migrating band (band 1) and a few faint bands around band 4 in the absence of sorbitol treatment (Supplementary Fig. S2, lane 3). After sorbitol treatment, intensity of the bands around band 4 increased whereas that of band 1 decreased (Supplementary Fig. S2, lane 4). Based on these results, we identified bands 1, 2, 3 and 4 as unphosphorylated CNOT2, phosphorylated CNOT2 at Ser126 (sorbitol-independent), phosphorylated CNOT2 at Ser101 or Ser165 (sorbitol-dependent) and phosphorylated CNOT2 at Ser101 or Ser165 (sorbitol-dependent) together with Ser126 phosphorylation (sorbitol-independent), respectively (Fig. 2B, 2A schematic representation of immunoblot images). In addition, the results of the triple mutant S101,126,165A indicated that there are other phosphorylation sites. Note that the differences in the fastest migrating bands between WT (Fig. 2B, lane 1) and the other samples (Fig. 2B, lanes 2–8) are due to a disorder of electrophoresis that sometimes occurs in Phostag-containing gel. Indeed, the fastest migrating bands are comparable between CNOT2 WT and the CNOT2 triple mutant S101,126,165A both in the absence or the presence of sorbitol (Supplementary Fig. S2, lanes 1–4). We performed additional experiments and confirmed that the fastest migrating band in CNOT2 WT-expressing cells without sorbitol treatment is similar with that in CNOT2 S101A-expressing cells (Supplementary Fig. S2, lanes 5–8).

(A) Alignment of CNOT2 amino acid sequences in various organisms and the consensus sequence for MK2, MAPK or CDK substrates. Asterisks indicate putative phosphorylation sites. (B) HEK293T cells transfected with vectors expressing the indicated CNOT2 constructs were treated with (+) or without sorbitol (-). Cell lysates were immunoprecipitated using anti-FLAG antibody. The Anti-FLAG immunoprecipitates were analysed by Phos-tag SDS-PAGE, followed by immunoblotting using anti-FLAG antibody. A schematic representation of immunoblot images in CNOT2 WT treated with (+) or without sorbitol (-) is shown at the bottom. (C) HEK293T cells transfected with vectors expressing the indicated CNOT2 constructs were treated with (+) or without sorbitol (-). Cell lysates were immunoprecipitated with anti-FLAG antibody. Immunoprecipitates (IP) and lysates were analysed by immunoblot. Anti-FLAG antibody detects exogenously expressed CNOT2. Immunoblots for phospho-p38MAPK was used to monitor the presence of osmotic stress-induced response (bottom). (D) HEK293T cells were transfected with the indicated constructs. Cell lysates were immunoprecipitated using anti-FLAG antibody. IPs and lysates were analysed by immunoblot. Anti-FLAG antibody and anti-Myc antibody detect exogenously expressed FLAG-CNOT2 and Myc-p38MAPK-AS (constitutively active p38MAPK), respectively. (E) HeLa cells were pretreated with inhibitors for 30 min and then treated with sorbitol for an additional 30 min. Cell lysates were analysed by immunoblot. SP: SP600125 (JNK inhibitor), SB: SB203580 (p38MAPK inhibitor), MK2i: (MK2 inhibitor). (F) An *in vitro* kinase assay was performed by incubating recombinant MK2 and indicated CNOT2 fragments in the absence (-) or presence (+) of ATP. Reaction products were analysed by immunoblot using phospho-CNOT2 S101 antibody (upper) and CBB staining (lower). A schematic representation of CNOT2 fragments is shown on the right.

To confirm that CNOT2 is phosphorylated at Ser101, we generated an antibody that recognizes Ser101-phosphorylated CNOT2 (hereafter, phospho-CNOT2 S101 antibody) by immunizing a rabbit with the phosphorylated peptide (see the Material and Methods). We confirmed that this antibody specifically reacted to the phosphorylated peptide by enzyme-linked immunosorbent assay (Supplementary Fig. S3A). The antibody detected a protein corresponding to endogenous CNOT2 after osmotic stress, which was blocked by the phosphorylated peptide, but not by unphosphorylated peptides (Supplementary Fig. S3B and C). We expressed FLAG-tagged CNOT2 WT and mutants possessing a single mutation S101A, S126A, or S165A in HEK293T cells, and prepared lysates before and after osmotic stress. Immunoblot analysis of anti-FLAG immunoprecipitates of the lysates revealed that the band intensity of the protein detected by phospho-CNOT2 S101 antibody increased after osmotic stress in immunoprecipitates of cells expressing CNOT2 WT, while no protein bands were detected in those expressing a single mutant S101A (Fig. 2C, lanes 3–6 in the top panel). Osmotic stress-induced CNOT2 S101 phosphorylation was not influenced by a single mutation S126A or S165A, though CNOT2 S101 phosphorylation in the absence of osmotic stress was reduced (Fig. 2C, lanes 7–10 in the top panel). Because amino acid sequences surrounding Ser126 or Ser165 correspond to a consensus target motif for MAPK- or CDK-mediated phosphorylation (Fig, 2A), we performed immunoblotting using the antibody against phosphorylated MAPK or CDK substrates that specifically detects phospho-serine in the motif. The antibody detected CNOT2 WT and single mutants S101A and S165A (Fig. 2C, lanes 3–6, 9 and 10 in second panel from the top), but not the single mutant S126A (Fig. 2C, lanes 7 and 8 in second panel from the top). In contrast to CNOT2 S101 phosphorylation (Fig. 2C, the top panel), the intensities of the detected bands were similar with and without sorbitol treatment (Fig. 2C, second panel from the top). These data suggest that Ser126 in CNOT2 is phosphorylated in a manner independent of osmotic stress.

We found that expression of constitutively active p38MAPK (p38MAPK-AS) [45] augmented phosphorylation of CNOT2 at Ser101 (Fig. 2D, lanes 3 and 4 in the top panel), while no protein bands were detected in a single mutant S101A (Fig. 2D, lanes 5 and 6 in the top panel). The CNOT2 S101 phosphorylation augmented by p38MAPK-AS was not influenced by a single mutation S126A, as in the case of osmotic stress-induced CNOT2 S101 phosphorylation (Fig. 2D, lanes 7 and 8 in the top panel). Furthermore, osmotic stress-induced CNOT2 S101 phosphorylation was suppressed by inhibitors of p38MAPK or MK2 [46] (Fig. 2E, lanes 4 and 5, respectively). These data suggest that MK2 phosphorylates CNOT2 at Ser101. Indeed, an *in vitro* kinase assay of MK2 using CNOT2N (amino acids 1–349 with or without S101A mutation) or CNOT2C (amino acid 350–540) fragments as substrates revealed that MK2 directly phosphorylates CNOT2 at Ser101 (Fig. 2F). We found that CNOT2 phosphorylation at Ser101 reached a peak around 1 h after osmotic stress and decreased afterwards (Supplementary Fig. S3D). Furthermore, phosphorylation of CNOT2 at Ser101 was also induced after anisomycin treatment, UV irradiation, and IL-1 stimulation (Supplementary Fig. S3E). These stimuli induced phosphorylation of p38MAPK, suggesting that CNOT2 phosphorylation at Ser101 is induced by extra- or intracellular stimuli that activate the p38MAPK pathway (Supplementary Fig. S3E).

### CNOT2-suppressed cells are sensitive to osmotic stress

**Figure 3.**
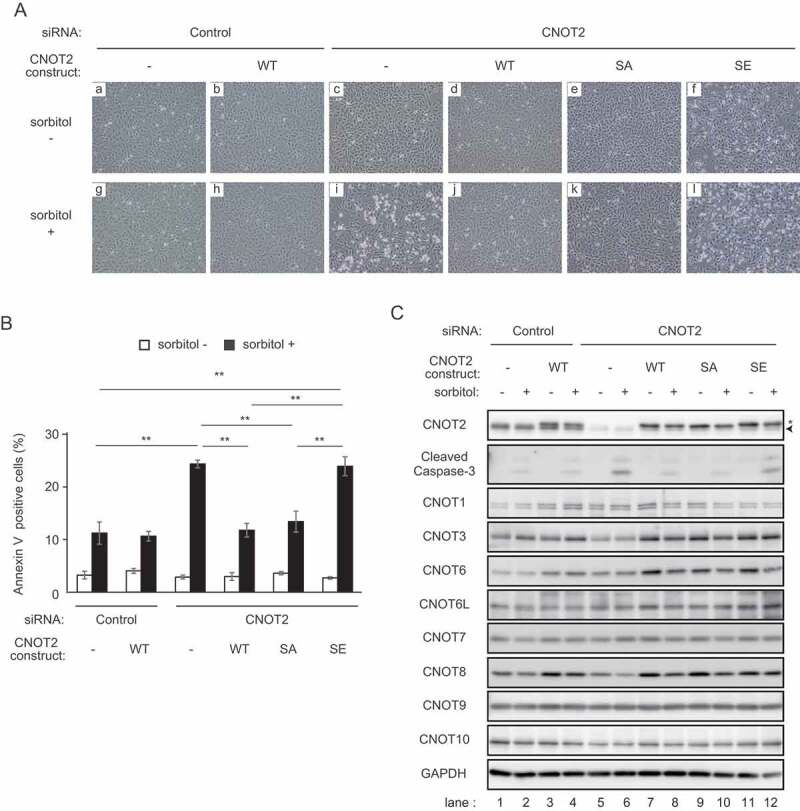
Phospho-mimic CNOT2 renders cells sensitive to osmotic stress.

**Figure 4.**
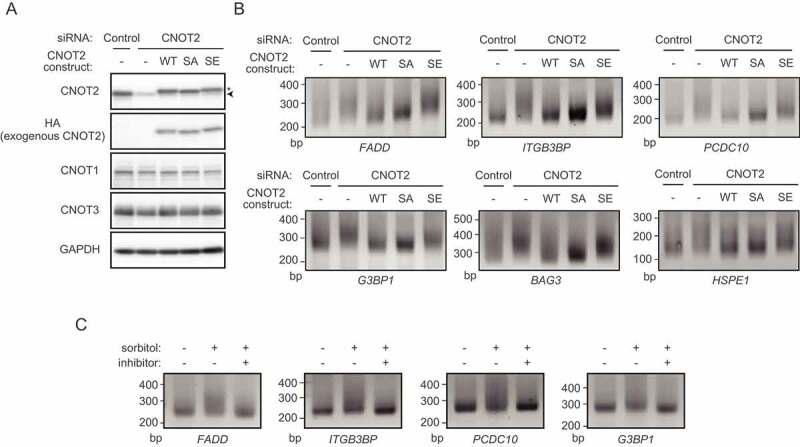
Insufficient poly(A) shortening in HeLa cells expressing phospho-mimic CNOT2.

We found that suppression of CNOT2 by introduction of siRNA against CNOT2 rendered HeLa cells much more sensitive to osmotic stress than control siRNA-treated cells, as indicated by an increased number of round and floating cells (Fig. 3A, compare panels g and i). We detected more Annexin V-positive cells among CNOT2-suppressed cells than control siRNA-treated cells after osmotic stress (Fig. 3B). Furthermore, a cleaved form of caspase 3 was more prominent in CNOT2-suppressed cells than control siRNA-treated cells upon osmotic stress (Fig. 3C, the second from the top, compare lanes 2 and 6). We confirmed that CNOT2 expression was efficiently reduced by siRNA transfection (Fig. 3C, top panel). Increase of Annexin V-positive cells and cleavage of caspase 3 are generally observed in apoptotic cells, suggesting that HeLa cells died by apoptosis. Note that CNOT3 and CNOT8 were marginally decreased upon CNOT2 knockdown. Similar results were obtained previously [16], but not so prominently as in *Drosophila* S2 cells [24]. When we re-introduced HA-tagged CNOT2 WT in CNOT2-suppressed HeLa cells using retrovirus, sensitivity to stress was comparable to that of control HeLa cells, as indicated by the absence of round and floating cells (Fig. 3A, compare panels g, h and i). Consistently, the number of Annexin V-positive cells and cleavage of caspase 3 after sorbitol treatment was comparable in CNOT2-suppressed cells that were reintroduced with HA-tagged CNOT2 WT and control siRNA-treated cells (Fig. 3B, 3C, the second from the top, compare lanes 2 and 8). Exogenous expression of HA-tagged CNOT2 WT in control HeLa cells did not have obvious effects on stress sensitivity before or after sorbitol treatment (Fig. 3A, panels a, b, g and h; 3B, C lanes 1–4). Note that the level of exogenously expressed HA-tagged CNOT2 WT was comparable to that of endogenous CNOT2 (Fig. 3C, top panel). Next, we expressed CNOT2 mutants mimicking hypo-phosphorylated and phosphorylated forms in CNOT2-suppressed HeLa cells to examine the effect of CNOT2 phosphorylation on stress sensitivity. While osmotic stress-induced phosphorylation of CNOT2 at Ser101 was confirmed by immunoblotting using phospho-CNOT2 S101 antibody, the results of Phos-tag SDS PAGE suggested that osmotic stress also induced phosphorylation of CNOT2 at Ser165 (Fig. 2B). Thus, we used the double mutant S101,165A (hereafter, the CNOT2 SA mutant) and a mutant in which stress-dependent phosphorylation sites in CNOT2 (both Ser101 and Ser165) were substituted with Glu in combination (hereafter, the CNOT2 SE mutant). We obtained similar results in cells expressing the HA-tagged CNOT2 SA mutant when compared to cells expressing HA-tagged CNOT2 WT (Fig. 3A, panels d, e, j and k; 3B, C lanes 7–10). In contrast, expression of the HA-tagged CNOT2 SE mutant in CNOT2-suppressed HeLa cells did not alter or may have slightly enhanced the stress sensitivity (Fig. 3A, panels c, f, i and l; 3B, C lanes 5, 6, 11 and 12). Again, levels of exogenously expressed HA-tagged CNOT2 SA or CNOT2 SE were comparable to that of endogenous CNOT2 (Fig. 3C, top panel). Furthermore, expression of CCR4-NOT complex subunits was comparable among HeLa cells expressing HA-tagged CNOT2 WT, SA or SE under CNOT2 knockdown conditions (Fig. 3C, the third panel from the top to the bottom panel). We confirmed that apoptosis is induced in both CNOT2-suppressed HeLa cells and those expressing the HA-tagged CNOT2 SE mutant by treating cells with a caspase inhibitor (Supplementary Fig. S4). These results suggested that phosphorylated CNOT2 fails to maintain cell survival under osmotic stress.

(A) HeLa cells were infected with retrovirus expressing mock (-) or the indicated CNOT2 constructs (WT, SA, or SE). They were subsequently transfected with control siRNA or siRNA against CNOT2. SiRNA-transfected cells were treated with (+, g-l) or without (-, a-f) sorbitol for 6 h. Representative images of the cells are shown. Round and floating cells increased in number after sorbitol treatment in CNOT2 siRNA-transfected cells that were infected with mock (-) and CNOT2 SE retrovirus (compare panels c and i, f and l). (B) Annexin V staining was performed to quantify the number of apoptotic cells in (A) (see the Materials and Methods). The graph shows percentages of annexin V-stained cells. Values represent means ± standard errors of means (n = 3). One-way ANOVA with Bonferroni/Dunnett’s multiple comparisons post hoc test was performed. **P < 0.01. (C) Lysates were prepared from HeLa cells treated as in (A) (sorbitol treatment for 6 h) and analysed by immunoblot. Lower bands indicated by an arrowhead and upper bands indicated by an asterisk correspond to endogenous CNOT2 and exogenously expressed HA-tagged CNOT2, respectively (top panel, please also see Fig. 4A).

(A) HeLa cells were infected with retrovirus expressing mock (-) or the indicated CNOT2 constructs (WT, SA, or SE). They were subsequently transfected with control siRNA or siRNA against CNOT2. Lysates were prepared from these cells and analysed by immunoblot. Lower bands indicated by an arrowhead and upper bands indicated by an asterisk correspond to endogenous CNOT2 and exogenously expressed HA-tagged CNOT2, respectively (the top panel). (B) Total RNA was prepared from cells shown in (A). Poly(A) tail lengths of mRNAs were analysed using the extracted total RNA (see the Materials and Methods). (C) HeLa cells were pretreated with (+) or without p38MAPK inhibitor (-) for 30 min. Subsequently, cells were treated with (+) or without sorbitol (-) for 30 min. Poly(A) tail lengths of mRNAs were analysed as in (B).

### The CCR4-NOT complex containing phosphorylated CNOT2 has less deadenylase activity

To examine whether CNOT2 phosphorylation influences deadenylase activity, we examined the mRNA poly(A) tail length. In these experiments, we prepared five cell types, HeLa cells transfected with control siRNA (1) or CNOT2 siRNA (2), and CNOT2 siRNA-transfected HeLa cells expressing HA-CNOT2 WT (3) or HA-CNOT2 SA (4) or HA-CNOT2SE (5). We confirmed that endogenous CNOT2 was efficiently reduced by transfection of CNOT2 siRNA and levels of exogenously expressed HA-tagged CNOT2 were similar with that of endogenous CNOT2 (Fig. 4A). After extracting total RNA from the cells, we performed poly(A) tail analyses (see the Materials and Methods) of mRNAs whose products are relevant to cell death, such as *Hspe1, Itgb3bp, Pdcd10, G3BP1, Bag3* and *Fadd* mRNAs. We found that their poly(A) tails were significantly longer in both CNOT2-suppressed HeLa cells and those expressing the CNOT2 SE mutant (Fig. 4B). On the other hand, polyA tail lengths of transcripts in HeLa cells expressing the CNOT2 WT or the SA mutant were comparable to those of control cells (Fig. 4B). Consistent with a previous report showing that the CCR4-NOT complex had no influence on polyA tails of mitochondrial mRNAs [47], poly(A) tails of *CYB* and *ND2* mRNAs were little affected in the absence of CNOT2 or the presence of CNOT2 SE (Supplementary Fig. S5). We also found that polyA tail lengths of *HDAC1* and *RPLP0* mRNAs were comparable among control HeLa cells, CNOT2-suppressed HeLa cells, and those expressing the CNOT2 SE mutant (Supplementary Fig. S5). Potentially, all mRNA species are targeted by the CCR4-NOT deadenylase [47]. On the other hand, major target mRNAs of the CCR4-NOT complex vary depending on tissues and cell types [11,13,14,19,20,23,48,49]. Therefore, polyA lengths of *HDAC1* and *RPLP0* mRNAs were hardly affected by suppression of the CCR4-NOT complex under our experimental conditions. Importantly, poly(A) tail lengths of *Hspe1, Itgb3bp, Pdcd10, G3BP1, Bag3* and *Fadd* mRNAs were elongated after sorbitol treatment (Fig. 4C). The elongation was not observed in the presence of p38MAPK inhibitor, further suggesting that sorbitol-induced CNOT2 phosphorylation is relevant to a reduction of CCR4-NOT deadenylase activity (Fig. 4C).

**Figure 5.**
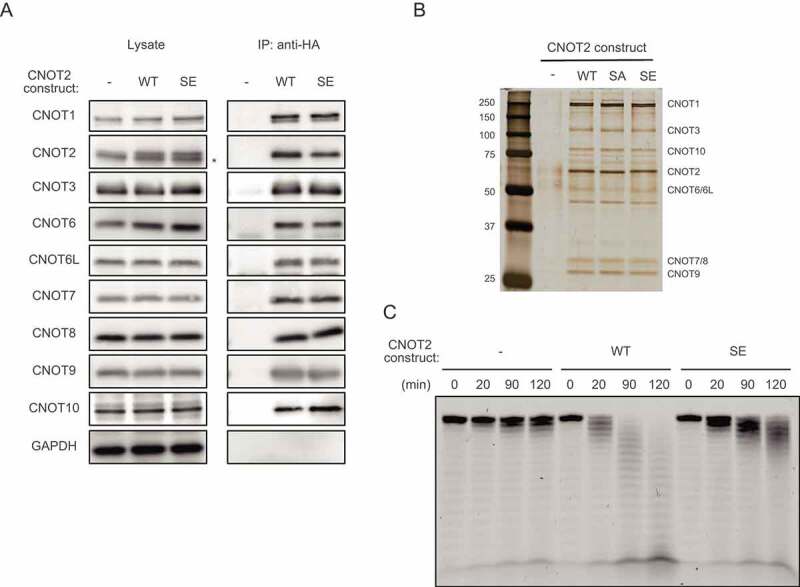
(A) Lysates were prepared from HeLa cells expressing HA-CNOT2 WT (WT) or HA-CNOT2 SE (SE) and immunoprecipitated using anti-HA antibody. The anti-HA immunoprecipitates (IP) and cell lysates were analysed by immunoblot. HeLa cells infected with control retrovirus (-) were used as controls. An asterisk indicates endogenous CNOT2. Please see Supplementary Fig. S6B for results of HA-CNOT2 SA. (B) Lysates were prepared from control HeLa cells (-) and HeLa cells expressing HA-CNOT2 (WT, SA or SE) and immunoprecipitated using anti-HA antibody. The anti-HA immunoprecipitates were analysed by silver staining. CNOT complex subunits predicted from molecular masses (but not identified by mass spectrometry) are indicated. (C) IPs prepared as in (A) were incubated with 5ʹ-labelled poly(A) RNA for the indicated times. Reaction products were then analysed on a denaturing gel.

To understand the reason for insufficient poly(A) shortening in cells expressing the CNOT2 SE mutant, we performed an *in vitro* deadenylation assay. We purified the CCR4-NOT complex by immunoprecipitation using anti-HA antibody from HeLa cells and those expressing HA-tagged CNOT2 WT or the CNOT2 SE mutant. Consistent with comparable complex assembly before and after sorbitol treatment (Supplementary Fig. S1), complex subunits were similarly co-precipitated with CNOT2 WT and the CNOT2 SE mutant (Fig. 5A). We also performed silver staining of the anti-HA immunoprecipitates and confirmed that co-purified CNOT complex subunits and other proteins were comparable between the complex containing CNOT2 WT and that containing CNOT2 SE (Fig. 5B). For the deadenylase reaction, we added a synthetic RNA labelled with fluorescein to the anti-HA immuneprecipitates as a substrate. We analysed reaction products on denaturing gels. The deadenylation reaction in the CCR4-NOT complex containing CNOT2 SE was less efficient than in that containing CNOT2 WT (Fig. 5C). On the other hand, the CCR4-NOT complex containing CNOT2 SA showed activity similar to that containing CNOT2 WT (Supplementary Fig. S6A). Again, we confirmed that CNOT complex subunits and co-purified proteins were comparable between the two conditions (Fig. 5B and Supplementary Fig. S6B).

## Discussion

Phosphorylation of proteins activates or suppresses their functions, and consequently modulates many biological processes. We found that extracellular stress induces phosphorylation of the CNOT2 subunit of the CCR4-NOT complex via the p38MAPK-MK2 pathway. This phosphorylation event attenuates the deadenylase activity of the complex and influences cellular responses. Yak1-mediated phosphorylation of Caf1 affects cell growth in response to glucose limitation, but it was not clear whether Caf1 deadenylase activity is affected [50]. This is the first evidence that activity of the CCR4-NOT deadenylase is directly regulated by protein kinases. MK2-mediated phosphorylation of ZFP36 family proteins leads to stabilization of mRNAs [37–39], indicating that both deadenylase activity and RBP function are regulated for mRNA stability in response to extra- or intracellular stimuli. Given that the CCR4-NOT complex is one of the major effectors of ZFP36 family protein-mediated mRNA decay, MK2-induced CNOT2 phosphorylation may function as a fail-safe mechanism to regulate mRNA turnover of ZFP36 family protein targets.

CNOT2 is phosphorylated in the absence of osmotic stress (Fig. 2C). Proliferating cells are exposed to intracellular signals such as production of reactive oxygen species (ROS). ROS activates the p38MAPK pathway [51], suggesting that CNOT2 S101 is phosphorylated in the absence of osmotic stress. However, the extent of CNOT2 S101 phosphorylation in the absence of osmotic stress was obviously low, compared to that of osmotic stress-induced CNOT2 phosphorylation (Fig. 2C). Consequently, we observed similar effects on poly(A) lengths in HeLa cells expressing HA-tagged CNOT2 WT and those expressing HA-tagged CNOT2 SA without stress (Fig. 4B). We observed that CNOT2 S101 phosphorylation declined 2 h after sorbitol treatment and remained unphosphorylated to a later time point (Supplementary Fig. S3D). Dephosphorylation of CNOT2 will resume deadenylase activity, leading to poly(A) tail shortening of transcripts. We propose that dephosphorylation of CNOT2 is important to terminate cellular signals activated by extra- or intracellular stresses so that cells return to a normal state. While an activity of exogenously expressed HA-CNOT2 WT is regulated by phosphorylation and dephosphorylation, HA-CNOT2 SE functions as a continuously phosphorylated molecule. We think that this difference is relevant to the different effect of poly(A) length between HA-CNOT2 WT and HA-CNOT2 SE in the absence of stress (Fig. 4B). Finally, expression of CNOT2 SE does not influence cell viability without osmotic stress, but reduces the deadenylase activity and simply renders cells sensitive to stress (Fig. 3). These data suggest that suppression of deadenylase activity though CNOT2 phosphorylation does not actively promote cell death. Consistent with this, a viability of CNOT2 siRNA-transfected HeLa cells in the absence of osmotic stress was comparable with that of control siRNA-transfected HeLa cells (Fig. 3).

CNOT2 is also phosphorylated by homeodomain-interacting protein kinases (HIPKs) [52]. Non-phosphorylatable or phospho-mimic CNOT2 mutants at five HIPK2-dependent phosphorylation sites show comparable effects on mRNA decay with CNOT2-WT. As CNOT2 Ser101 (a non-HIPK2 target), but not Ser165 (HIPK2 target) is critical for regulation of the CCR4-NOT complex, MK2-mediated phosphorylation of CNOT2 at Ser101 is an important mechanism for cells to deal with environmental stress. It should be noted that the CCR4-NOT complex containing phospho-mimic CNOT2 still had slight, but evident deadenylase activity (Fig. 4). In this study, we focused on CNOT2 phosphorylation, based on the mobility shift in SDS-polyacrylamide gels. It is possible that yet unidentified phosphorylation sites of CNOT2 are also involved in the regulation of deadenylase activity (Supplementary Fig. S2). Furthermore, other subunits of the CCR4-NOT complex may be similarly phosphorylated and may also be responsible for regulation of deadenylase activity. Indeed, high-throughput proteomic analyses have identified phosphorylation of other CCR4-NOT complex subunits [43]. Some phosphorylation events appear relevant to extracellular signals. Phosphorylation studies of other subunits will help illuminate extracellular signal-dependent regulation of the deadenylase activity.

Cellular stresses causing ribosome collisions such as anisomycin induce activation of the p38MAPK and JNK pathways and affect cell viability [53]. CNOT2 is phosphorylated at Ser101 by anisomycin treatment (Supplementary Fig. 3E), suggesting that translation stress influences on mRNA stability. It is intriguing to examine whether ribosome collision-induced activation of p38MAPK-MK2 signalling involves CCR4-NOT complex-mediated mRNA decay via CNOT2 phosphorylation to regulate cell fate.

The CCR4-NOT complex functions in a variety of biological processes in higher organisms, such as embryogenesis, tissue development, and cellular and tissue homoeostasis. Since the CCR4-NOT complex basically targets a lot of poly(A) mRNAs [47,54], context-dependent regulation of its deadenylase activity should be vitally important in each process. Several post-translational modifications of CCR4-NOT complex subunits have been identified and some of them contribute to global regulation of mRNA abundance and cellular responses [52,54]. Our results demonstrate an important relationship between kinase signalling and CCR4-NOT complex deadenylase activity that provides dynamic regulation of mRNA deadenylation in response to extra- or intracellular environmental changes.

## Materials and methods

### Cells and reagents

HeLa (RCB0007) and HEK293T (RCB2202) cells were provided by the RIKEN BRC through the National Bio-Resource Project of MEXT, Japan. They were grown in Dulbecco’s modified Eagle’s medium with 10% FBS and 100 U/mL penicillin/streptomycin. Anisomycin was purchased from Sigma. SP600125, SB203580, and MK2 inhibitor III were from MERCK Millipore. Sorbitol was from WAKO. Recombinant Human IL1β/IL-1F2 was from R & D Systems. Concentrations used in this study are as follows: Anisomycin (10 μM), sorbitol (0.5 M) and recombinant Human IL1β/IL-1F2 (10 ng/mL). Inhibitors, SP600125 (50 μM), SB203580 (60 μM), or MK2 inhibitor III (150 μM) were added to cells 30 min before sorbitol treatment.

### Antibodies

Antibodies against CNOT3, CNOT6, CNOT6L, CNOT8 and CNOT9 were used as previously described [14,20]. Antibodies against CNOT1 (14,276-1-AP) and CNOT10 (A304-899A) were purchased from Proteintech and Bethyl Laboratories, respectively. Antibody against CNOT7 (H00029883-M01) was from Abnova. Antibodies against phosphorylated MAPK or CDK substrates (#2325), p38MAPK (#8690), phospho-p38MAPK (#4511), CNOT2 (#34,214) (for immunoblot), JNK (#9252), phospho-JNK (#4671), and cleaved caspase-3 (#9664) were from Cell Signalling Technologies. An antibody against FLAG-peptide (F1804) was from Sigma. Antibodies against GAPDH (M171-3) and HA (M180-3) were from MBL. Antibody against Myc-epitope (011–21,874) and normal mouse immunoglobulin G (IgG) (140–09511) were from WAKO. We generated polyclonal antiserum against Ser101-phosphorylated CNOT2 by immunizing a rabbit with the synthetic peptide CQLNRSLS(-PO_4_)QGTQL coupled to KLH by the N-terminal cysteine. After depleting antibodies reactive with the corresponding non-phosphorylated peptide, phospho-specific antibodies were affinity-purified with an immunizing peptide-conjugated column. Immunizations, enzyme-linked immune-sorbent assays, and serum collection were performed at Eurofin Genomics.

### Enzyme-linked immune-sorbent assays

Non-phosphorylated or phosphorylated peptides used for generation of phospho-CNOT2 S101 antibody were dissolved in phosphate-buffered saline (PBS) (5 μg/mL). Peptide solution was aliquoted into 96-well plates (100 μL/well) and incubated for 2 h at room temperature. Wells were washed with buffer A (PBS containing 0.2% Tween 20) three times. Then, buffer A (100 μL) was added to wells and incubated for 2 h at room temperature again. Antiserum or purified antibody was diluted with buffer B (PBS containing 0.05% Tween 20) from 1:1000 to 1:128,000. Diluted solutions (100 μL) were put in wells and incubated for 1 h at room temperature. Wells were washed three times with buffer B. Secondary antibody (Goat Anti-Rabbit IgG (Fab’)2-HRP conjugated, 55,676, MP Biomedicals) diluted with buffer B at 1:5000 (100 μL) was added to wells and incubated for 1 h at room temperature. Wells were washed with buffer B three times and incubated for 20 min at room temperature after addition of 100 μL of 50 mM citrate-phosphate buffer (pH 5.0) containing o-Phenylenediamine (0.4 mg/mL) and 0.006% hydrogen peroxide. The reaction was stopped by adding 100 μL of 1 M sulphuric acid and the optical density was measured at 490 nm.

### Immunoprecipitation and immunoblot

Cells were washed with PBS, and were lysed with TNE buffer (50 mM Tris–HCl [pH 7.5], 150 mM NaCl, 1 mM EDTA, 1 mM phenylmethylsulfonylfluoride, 10 mM NaF, 10 mM -glycerophosphate 50, 1% Nonidet P-40) containing PhosSTOP phosphatase inhibitors (4,906,845,001, Roche). Cell lysates were subjected to immunoprecipitation using mouse monoclonal antibody against CNOT2 [16] (1 μg) bound to Protein G Sepharose (GE Healthcare). For immunoprecipitation of FLAG-tagged or HA-tagged proteins, we used anti-FLAG antibody-conjugated agarose (A2220, Sigma) or anti-HA antibody-conjugated agarose (A2095, Sigma), respectively. Immunoprecipitates or equal amounts of proteins were resolved on SDS-polyacrylamide gels and transferred to Immobilon-P (Millipore). Membranes were blocked with 3% non-fat dry milk (Morinaga, Tokyo, Japan) in Tris-buffered saline containing 0.05% Tween 20 (TBST) for 2 h at RT. Membranes were incubated with the indicated primary antibodies overnight at 4°C. We used Can Get Signal® Immunoreaction Enhancer Solution (NKB-101, TOYOBO, Osaka, Japan) for antibody dilution, except for CNOT2 S101-P antibody. CNOT2 S101-P antibody was diluted in 3% non-fat dry milk in TBST. Membranes were washed with TBST, 3x for 10 min at RT, and then incubated with Mouse IgG HRP-Linked Whole Ab or Rabbit IgG HRP-Linked Whole Ab (NA931 or NA934, Cytiva, Tokyo, JAPAN) as secondary antibodies for 1 h at RT. Again, membranes were washed with TBST, 3 times for 10 min at RT. We used a Western Lightning Plus ECL (Perkin Elmer, Waltham, MA, USA) for developing membranes, and images were detected on an Amersham Imager 600 (GE Healthcare). For peptide competition (Supplementary Fig. S3C), CNOT2 S101-P antibody (400 ng) was incubated with 2 μg of non-phosphorylated or phosphorylated peptide in 3% non-fat dry milk in TBST (0.5 mL) for 2 h and subsequently used for immunoblotting.

### UV irradiation

Cells at 80% confluence were exposed to UV-C (50 J/m^2^) with a FUNA-UV-linker (FS-800; FUNAKOSHI). After incubation in medium for the indicated times, cells were washed with PBS and were lysed with TNE buffer.

### Phos-tag gel electrophoresis

Cells were washed with buffer (20 mM HEPES-NaOH, pH 7.4, and 150 mM NaCl) and were lysed with TNE buffer containing PhosSTOP phosphatase inhibitors. Electrophoresis was performed in gels containing 5% polyacrylamide, 200 μM MnCl_2_, and 100 μM Phos-tag (Fig. 1B, 1C) and 5% polyacrylamide, 100 μM MnCl_2_, and 50 μM Phos-tag (Fig. 2B and Supplementary Fig. S2) (AAL-107, NARD Institute).

### Expression vectors and transient transfection

Human CNOT2 cDNA was inserted into pcDNA3.1 vector (ThermoFisher) or pMXs-puro vector. FLAG-tag or HA-tag sequences were included in PCR primers. CNOT2 mutants were generated using KOD mutagenesis Kits (SMK-101, TOYOBO). In pMXs-puro vectors, the CNOT2 nucleotide sequence has silent mutations to resist siRNA against CNOT2. We verified these expression constructs by sequencing. We obtained pcDNA-Myc-p38MAPK-AS from Dr Mutsuhiro Takekawa (Univ. of Tokyo). We used pcDNA3.1-FLAG-tagged CNOT2 constructs in Fig. 2B-D and Supplementary Fig. S2 and used pMXs-puro-HA-tagged CNOT2 constructs in Fig. 3, Fig. 4A, 4B, Fig. 5, and Supplementary Figs. S4-6. We used TransIT-LT1 (MIR-2300, MirusBio) when introducing expression vectors into HEK293T cells (Fig. 2). We mixed pcDNA3.1 plasmid vectors (2 μg or 1 μg each when introducing two vectors) with 6 μL of TransIT-LT1 reagent in 200 μL of OPTI-MEM (ThermoFisher). The transfection mix was incubated for 15 min at room temperature and added into HEK293T cells (5 × 10^5^ cells on 6-cm dish).

### Virus infection and siRNA transfection

Lentivirus for introduction of ecotropic receptors was produced by transfecting 293 FT cells (Thermo Fisher Scientific, R70007) with pLenti6/UbC/Slc7a1 (addgene #17,224) and packaging mix (Thermo Fisher Scientific, JPG0035) using Lipofectamine 2000 (Thermo Fisher Scientific, 11668-019). HeLa cells were transfected with the lentivirus and selected with blasticidin (10 μg/mL). Retroviruses were produced by transfecting Plat-E packaging cells (3.5 × 10^5^ cells on 6-cm dish) with 2 μg pMXs-puro vectors containing HA-tagged CNOT2 cDNAs using TransIT-LT1 transfection reagent (6 μL). Two days after transfection, cell culture supernatants containing the retroviruses (4 mL) were filtered (MILLEX GV 0.45 μm, Millipore) and polybrene (5 μg/mL, Sigma) was added. Resultant viral solutions (3.5 mL) were used for infection of HeLa cells expressing ecotropic receptors. Cells were seeded at 8.5 × 10^5^ cells on 10-cm dish the day before infection. Two days after retroviral infection, cells were diluted following trypsinization and cultured in the presence of puromycin (1 μg/mL, Sigma) for an additional 3 days to select infected cell populations, which were subsequently used for analyses. This condition makes exogenous HA-tagged CNOT2 expression comparable to endogenous CNOT2. SiRNA (100 *p*mol) was transfected into retrovirus-infected HeLa cells (5 × 10^5^ cells on 6-cm dish) with 9 μl of Lipofectamine RNAiMAX (Thermo Fisher Scientific). Cells were treated with sorbitol 2 days after siRNA transfection.

Sequences of siRNAs are as follows: Control siRNA, sense, 5ʹ-UUCUCCGAACGUGUCACGUTT-3ʹ, anti-sense 5ʹ-ACGUGACACGUUCGGAGAATT-3ʹ.

CNOT2 siRNA, sense, 5ʹ-CCACGUCACGCCAACAACAGG-3ʹ, anti-sense, 5ʹ-UGUUGUUGGCGUGACGUGGCU-3ʹ.

### Quantification of cell death

To quantify percentages of apoptotic cells, we used Annexin-V, which binds to externalized phosphatidylserine residues from the inner membrane of the cytoplasmic membrane during apoptosis. HeLa cells were collected following trypsinization and washed with cold PBS. Then, cells (1x10^6^ cells) were resuspended in 300 µL binding buffer. Cells were incubated with 5 µL Annexin V-FITC (Biolegend 640,905) in the dark for 15 min at room temperature. We obtained data regarding Annexin V-positive cells from at least 30,000 events using a FACS ARIA III (BD Biosciences). To analyse the data, we used FlowJo 10.3 software (Tree Star, OR, USA).

### Preparation of GST-fusion proteins

Human CNOT2 fragments, CNOT2N (1 to 350 aa, with or without a single mutation S101A) and CNOT2C (351 to 540 aa) were amplified with PCR and inserted into pGEX-6P1. GST-CNOT2C and GST-CNOT2N proteins were produced in the *E. coli* BL21-strain following isopropyl-β-D-thiogalactopyranoside induction and proteins were purified with glutathione-Sepharose 4B (GE Healthcare).

#### In vitro *kinase assay*

Recombinant GST-fused CNOT2 proteins (1 μg) were in kinase buffer (20 mM Tris-HCl, pH 7.4, 10 mM MgCl_2_, 3 mM MnCl_2_, 0.1 mM ATP) and mixed with 20 ng of active MK2 (SignalChem, M40-11 H) at 30°C for 30 min. The reaction was performed without ATP as a control reaction. After addition of SDS-PAGE sample buffer, reaction mixtures were incubated at 100 ℃ for 5 min and separated on 7.5% SDS-PAGE.

### Poly(A) tail assay

Poly(A) tail lengths of mRNAs were analysed using Poly(A) Tail-Length Assay Kits (Thermo Fisher Scientific), according to the manufacturer’s protocol. Briefly, total RNA (1 μg) was incubated with poly(A) polymerase in the presence of GTP (G) and ITP (I) to add a GI tail. cDNA was generated with PCR poly(A) test (PAT) universal primer and reverse transcriptase using GI-tailed RNA as a template. PCR amplification was performed with gene-specific and PAT universal primers and HotStart-IT Taq DNA polymerase. Gene-specific primers are as follows:

*FADD*, 5ʹ-gcaattctacagtttcttactgttttgtat-3ʹ, *PDCD10*, 5ʹ-tcttttaagaattataagccaaaagaatttac-3ʹ, *G3BP1*, 5ʹ-aagaaggaatgttactttaatattggactt-3ʹ, *HSPE1*, 5ʹ-agttctgaaatctttcgtcatgtaaataat-3ʹ, *ITGB3B*, 5ʹ-taatacatttcttcttggctctttaatgta-3ʹ, *BAG3*, 5ʹ-aattacccatcacataaatatgaaacatt-3ʹ, *CYB*, 5ʹ-actaagccaatcactttattgactccta-3ʹ, *ND2*, 5ʹ-cttaacctctacttctacctacgcctaa-3ʹ, *HDAC1*, 5ʹ-cctaattctgcaggtggaggtt-3ʹ, *RPLP0*, 5ʹ-tgtctgtggagacggattacac-3ʹ.

### Preparation of the CCR4-NOT complex and silver stain

Lysates were prepared from HeLa cells infected with mock retrovirus or those expressing HA-tagged CNOT2 constructs (WT, SA, or SE) using TNE buffer. Cell lysates (containing 30 mg of total protein) were immunoprecipitated using anti-HA antibody-conjugated agarose gels. Purified immunoprecipitates were used for silver staining or deadenylase reaction. Silver Stain 2 Kit (291–50,301, WAKO) was used according to the manufacturer’s protocol.

#### In vitro *deadenylation assay*

The purified CCR4-NOT complexes containing HA-tagged CNOT2 (WT, SA, or SE) in 50 μL nuclease buffer (50 mM Tris-HCl [pH 7.5], 150 mM NaCl, 2 mM MgCl_2_, 10% glycerol, 1 mM dithiothreitol) were incubated at 37 ℃ with synthesized RNA substrate (5ʹ-UCUAAAUAAAAAAAAAAAAAAAAAAAA-3ʹ, final concentration, 0.1 μM) labelled with fluorescein isothiocyanate at the 5ʹ-end. Reaction products (10 or 12.5 μL) were collected at the indicated times, mixed with an equal volume of 5% formaldehyde solution, and boiled at 95°C for 5 min. Final products were separated on a 7 M urea–25% polyacrylamide denaturing gel. The gel was analysed with an Amersham Imager 600 (GE Healthcare).

## Supplementary Material

Supplemental Material
