# Supplementary material for: Regulation of CCR4-NOT complex deadenylase activity and cellular responses by MK2-dependent phosphorylation of CNOT2
Source: RNA Biol. 2022 Feb 6;19(1):234–46. doi: 10.1080/15476286.2021.2021676 (PMC8820811; doi:10.1080/15476286.2021.2021676)
Supplement: Supplemental Material [file KRNB_A_2021676_SM1045.zip › supplementary/supplementary.pdf]

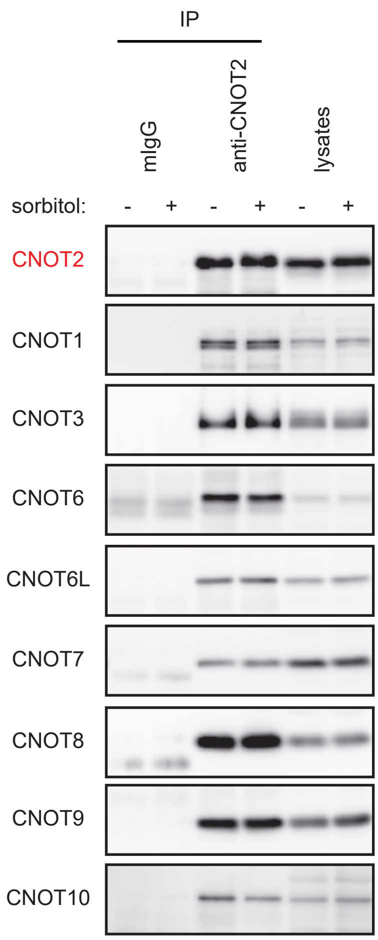

Supplementary Figure S1 Formation of the CCR4-NOT complex before and after sorbitol treatment

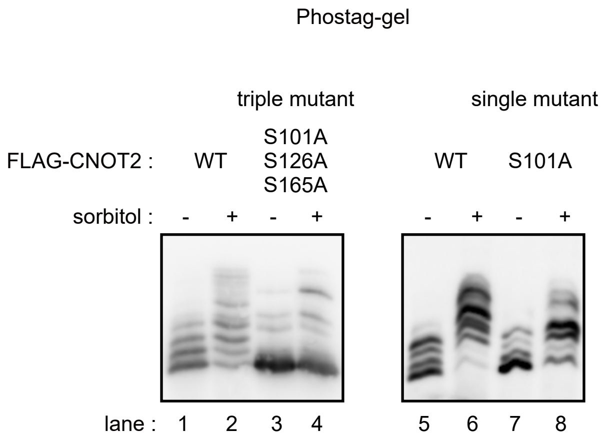

Supplementary Figure S2 Phostag SDS-PAGE analyses detect phosphorylation of CNOT2 at Ser101, 126 and 165

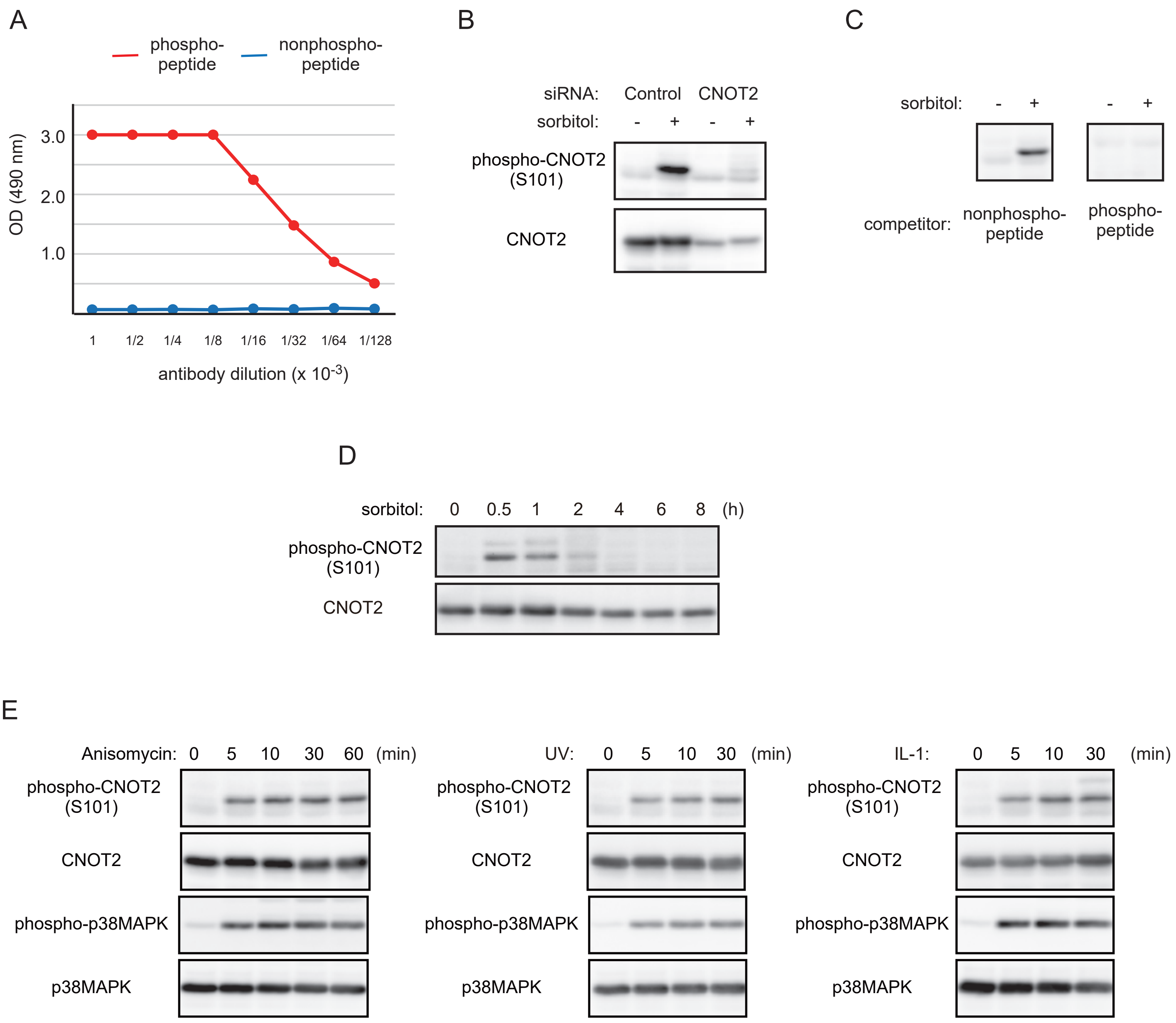

Supplementary Figure S3 Validation of phosphorylated CNOT2-specific antibody and phosphorylation of CNOT2 in response to various stimuli

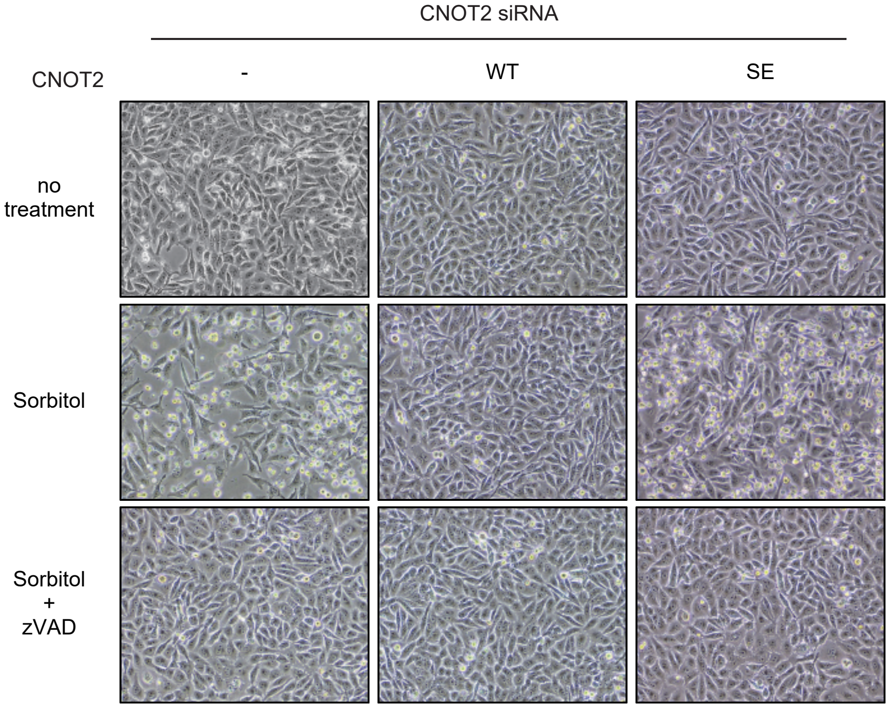

Supplementary Figure S4 HeLa cells lacking CNOT2 or expressing phospho-mimic CNOT2SE undergo apoptosis

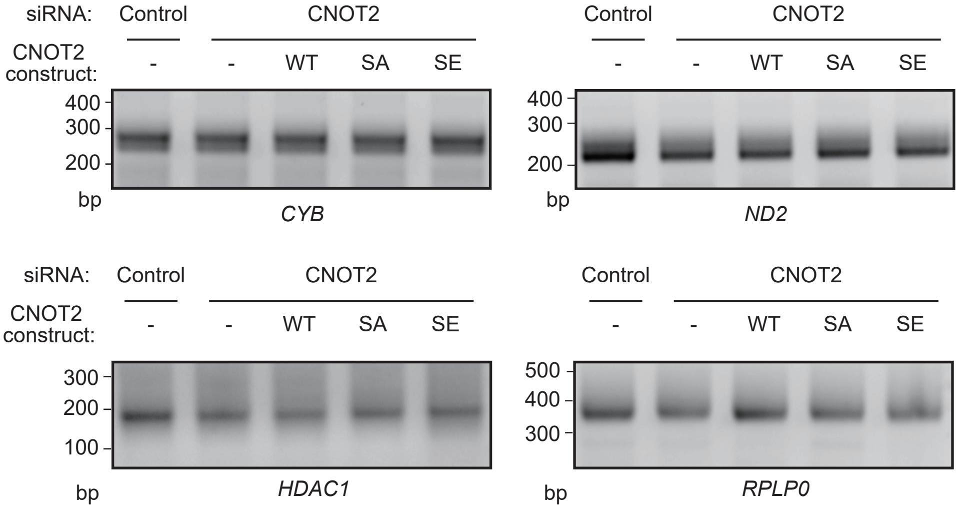

Supplementary Figure S5 PolyA lengths of several mRNAs are not influenced by expression of CNOT2 SE

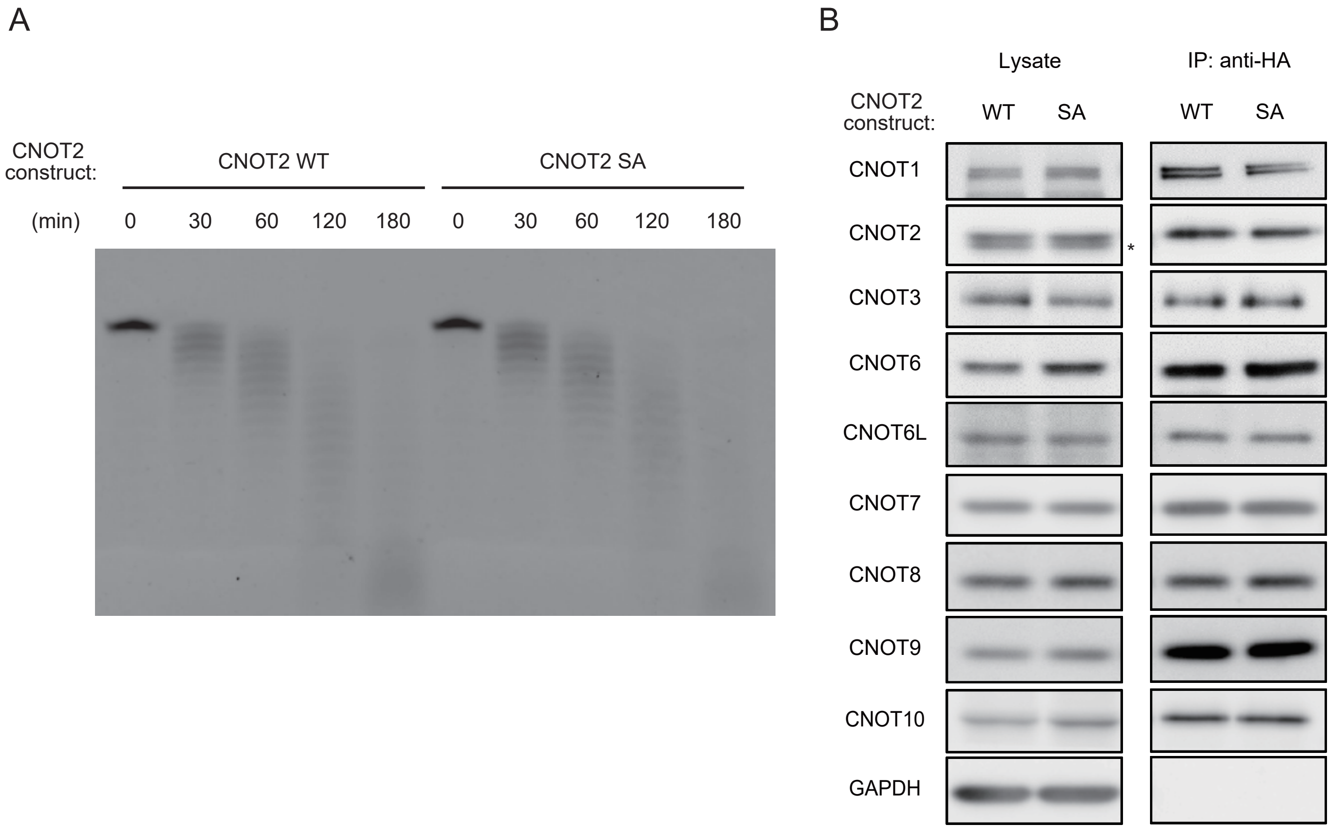

Supplementary Figure S6 Comparable deadenylase activity between CNOT2 WT and CNOT2 SA

Supplementary Figure legends

Supplementary Fig. S1

HeLa cells were treated with (+) or without (-) sorbitol for 30 min. Cell lysates were immunoprecipitated using normal mouse IgG or anti-CNOT2 mouse monoclonal antibody. Immunoprecipitates (IP) and cell lysates were analyzed by immunoblotting using the indicated antibodies. CNOT2 is shown in red to indicate a precipitated molecule.

Supplementary Fig. S2

HEK293T cells transfected with vectors expressing the indicated CNOT2 constructs were treated with (+) or without sorbitol (-). Cell lysates were immunoprecipitated using anti-FLAG antibody. The Anti-FLAG immunoprecipitates were analyzed by Phos-tag SDS-PAGE, followed by immunoblotting using anti-FLAG antibody.

Supplementary Fig. S3

(A) ELISA of phospho-CNOT2 S101 antibody. Phospho-CNOT2 S101 antibody was diluted as indicated. OD490 against the non-phosphorylated (blue line) and phosphorylated peptides (red line) was measured in each antibody solution. (B) HeLa cells transfected with control siRNA or siRNA against CNOT2 were treated with (+) or without sorbitol (-). Lysates were prepared 30 min after treatment and analyzed by immunoblot. (C) HeLa cells were treated with (+) or without sorbitol (-). Lysates were prepared 30 min after treatment. Immunoblot analysis was performed after incubating CNOT2 S101-P antibody with non-phosphorylated or phosphorylated peptide (see the Materials and Methods). (D, E) HeLa cells were treated with sorbitol, anisomycin, UV, or IL-1. Lysates were prepared at the indicated times after treatment and analyzed by

immunoblot.

Supplementary Fig. S4

HeLa cells expressing the indicated CNOT2 mutants were transfected with control siRNA or siRNA against CNOT2. SiRNA-transfected cells were treated with (+) or without (-) sorbitol in the presence of zVAD (40 μM) for 24 h. Representative images of cells are shown. Round and floating cells were observed in CNOT2 siRNA-transfected cells that were infected with mock (-) and CNOT2 SE retrovirus (left and right in the middle). Those cells were hardly detected in the presence of zVAD (left and right at the bottom).

Supplementary Fig. S5

HeLa cells were infected with retrovirus expressing empty vector (-) or the indicated CNOT2 constructs (WT, SA, or SE). They were subsequently transfected with control siRNA or siRNA against CNOT2 (see Fig. 4). Total RNA was prepared from the cells. Poly(A) tail lengths of mRNAs were analyzed using the extracted total RNA (see the Materials and Methods).

Supplementary Fig. S6

(A) Lysates were prepared from HeLa cells expressing HA-CNOT2 WT or HA-CNOT2 SA and immunoprecipitated using anti-HA antibody. The anti-HA immunoprecipitates were incubated with 5'-labeled poly(A) RNA for the indicated times (min). Reaction products were then analyzed on a denaturing gel. (B) Immunoprecipitates (IP) and lysates prepared in (A) were analyzed by immunoblot. An asterisk indicates endogenous CNOT2.
